# Identification of Hub Genes With Differential Correlations in Sepsis

**DOI:** 10.3389/fgene.2022.876514

**Published:** 2022-03-24

**Authors:** Lulu Sheng, Yiqing Tong, Yi Zhang, Qiming Feng

**Affiliations:** ^1^ Department of Emergency Medicine, Shanghai Jiao Tong University Affiliated Sixth People’s Hospital, Shanghai, China; ^2^ Biomedical Research Center, Institute for Clinical Sciences, Zhongshan Hospital, Fudan University, Shanghai, China

**Keywords:** WGCNA, differential correlation, regulatory network, biological analysis, sepsis

## Abstract

As a multifaceted syndrome, sepsis leads to high risk of death worldwide. It is difficult to be intervened due to insufficient biomarkers and potential targets. The reason is that regulatory mechanisms during sepsis are poorly understood. In this study, expression profiles of sepsis from GSE134347 were integrated to construct gene interaction network through weighted gene co-expression network analysis (WGCNA). R package DiffCorr was utilized to evaluate differential correlations and identify significant differences between sepsis and healthy tissues. As a result, twenty-six modules were detected in the network, among which blue and darkred modules exhibited the most significant associations with sepsis. Finally, we identified some novel genes with opposite correlations including ZNF366, ZMYND11, SVIP and UBE2H. Further biological analysis revealed their promising roles in sepsis management. Hence, differential correlations-based algorithm was firstly established for the discovery of appealing regulators in sepsis.

## Introduction

Sepsis and septic shock with subsequent multi-organ failure contribute to the leading causes of death among patients in adult intensive care unit (ICU), which are due to massive inflammatory responses to infection (Abe et al., 2020; Markwart et al., 2020). The incidence of sepsis is approximately 20 million cases each year with 30–50% high mortality in the United States (Fleischmann et al., 2016). Advances in understanding pathophysiology of sepsis reveal that it occurs not only with inflammation-related responses but also modifications in non-immunological pathways (Rello et al., 2017; Yu et al., 2021). Despite great improvement in surgery, pharmacological approaches and serum biomarkers including procalcitonin (PCT), C-reactive protein (CRP), lactate and cell-free DNA has been made in initial detection and therapy of sepsis, the incidence and mortality rates are still rising rapidly due to complexity of sepsis and lack of targeted drugs (Povoa et al., 2005; Saukkonen et al., 2008; Riedel et al., 2011; Rhee et al., 2015). Thus, novel risk genes and related regulatory networks need to be identified to illustrate sepsis etiology and direct researchers to develop effective therapeutic strategies.

Accumulating studies have used transcriptome data comprised of cellular components contents between disease and healthy tissues to decipher potential molecular mechanisms of sepsis (Zhang et al., 2020a; Fang et al., 2021; Yu et al., 2021). Meanwhile, most of these studies incorporated differentially expressed genes and gene correlation data to explore gene interaction networks, followed by enrichment analysis to clarify function of unknown genes (Balamuth et al., 2020; Zhai et al., 2020; Zhang et al., 2020b). Nevertheless, the biggest limitation is that the observed gene correlations may be redundant because they appeared in both two states, adding difficulties in the discovery of true causative genes. The solution to this problem comes to the identification of differential correlations referring to alterations of correlated patterns under different conditions (Ideker and Krogan, 2012; Li et al., 2015; Zhou et al., 2021). Currently, differential correlations in sepsis have been poorly understood, hence it is in urgent need to orchestrate network dynamics for identifying novel candidate genes.

**FIGURE 1:**
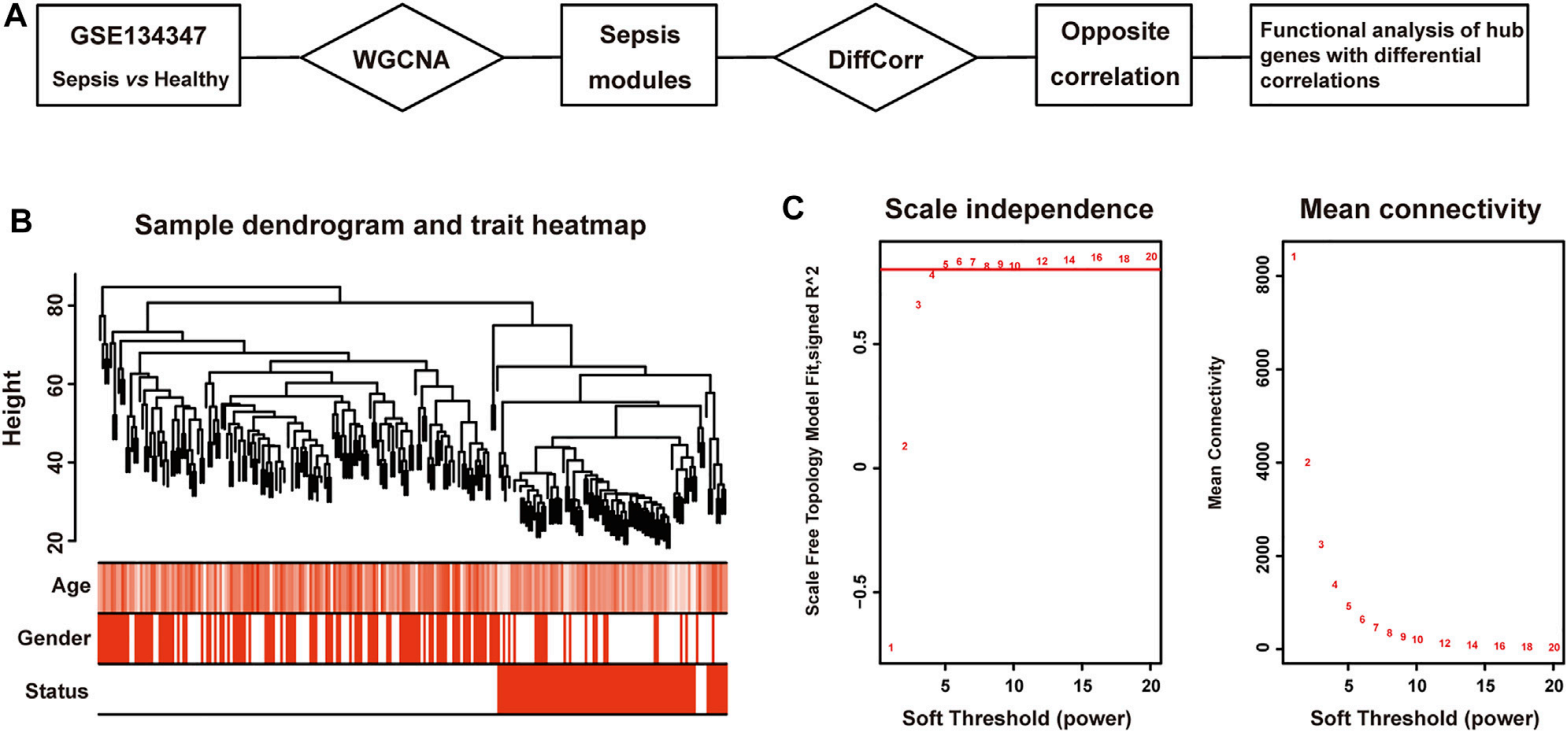
Clustering dendrogram of sepsis and healthy tissues. **(A)** The workflow of this study. **(B)** Clustering dendrogram of 156 patients with sepsis and 82 healthy subjects and trait heatmap. **(C)** The relationship between soft threshold (power) and network properties. Left panel: The relationship between soft-threshold (power) and scale-free topology. Right panel: The relationship between soft threshold (power) and mean connectivity.

Here, an *in silico* framework was proposed to identify hub genes with differential correlations in sepsis (Figure 1A). First, the networks of gene expression were constructed using weighted gene co-expression network analysis (WGCNA), which found correlated genes based on gene connectivity and formed gene modules (Ghazalpour et al., 2006; Yang et al., 2018). Twenty-six modules were detected in the network, among which blue and darkred modules exhibited the most significant associations with sepsis. Next, differential correlations of genes in these two modules were calculated and significant differences between sepsis and healthy tissues utilizing R package DiffCorr were identified. Finally, we identified some novel genes including ZNF366, ZMYND11, SVIP and UBE2H. Further biological analysis revealed their promising roles in sepsis management.

## Methods

### Sepsis Expression Profiles

The HTA2.0 microarray data of 156 patients with sepsis and 82 healthy subjects was downloaded from GEO with primary data accession number GSE134347 (https://www.ncbi.nlm.nih.gov/geo/query/acc.cgi?acc=GSE134347). Patient characteristics were described in (Scicluna et al., 2020).

### Co-Expression Network Construction

Screening of correlated gene pairs was performed by an R package WGCNA (Langfelder and Horvath, 2008). And step-by-step calculation was as follows:

Step 1 The Pearson correlation coefficient between gene *X*_*i*_ and *X*_j_ was calculated, and matrix *X* was converted into the correlation matrix *S* between genes:

$$S_{ij}=\left|cor\left(X_{i},X_{j}\right)\right|$$ (1)

Step 2 Weighted adjacency matrix aij was constructed and suitable soft-threshold power β was selected:

$$a_{ij}={\left|S_{ij}\right|}^{\beta }$$ (2)

Step 3 The degree of separation of nodes was calculated. The adjacency matrix is converted into unsigned topological overlap matrix (TOM) to calculate degree of intergenic dissimilarity (Zhang and Horvath, 2005), based on which genes were distributed in different modules:

$$TOM_{ij}=\frac{l_{ij}+a_{ij}}{\min\left(k_{i},k_{j}\right)+1-a_{ij}};l_{ij}=\sum_{u}a_{iu}a_{ju}$$ (3)

In the above formula, *l*_*ij*_ represented the sum of product of adjacency coefficients of all common adjacent genes of gene *i* and *j*. *a*_*ij*_ represented the adjacency coefficient between gene *i* and *j*. *k*_*i*_ represented synthesis of adjacency coefficients of gene *i* with all neighboring nodes. If *TOM*_*ij*_ was 0, it meant that gene *i* and *j* were isolated and not connected to all other genes. If *TOM*_*ij*_ was 1, it meant that these two genes were adjacent to all surrounding genes and were also connected to each other. In other words, *TOM* represented the similarity of genes, so the dissimilarity between genes could be calculated:

$$DistTOM_{ij}=1-TOM_{ij}$$ (4)

Step 4 *DistTOM*_*ij*_ was used for hierarchical clustering, and genes were divided into different co-expression modules (Li and Horvath, 2007). WGCNA adopted Dynamic Tree Cut Method to construct the cluster tree, which was a top-down merging algorithm. Through iteration and decomposition of gene clusters, stable gene clusters were eventually achieved (Langfelder et al., 2008). Here, the minimum module size was set as 30 to identify modules and draw dendrogram.

Step 5 Identify trait-related modules. Herein, trait was defined as disease. We defined gene significance (GS) as relationship between gene expression levels and disease. Moreover, module membership (MM) represented the degree of relationship between module feature genes and disease. The higher MM represented the higher correlation between modules and disease. At last, we identified two modules most relevant to disease. One module exhibited positive correlation and another negative correlation.

### Differential Correlation Evaluation

R package DiffCorr was utilized for the visualization and identification of differential correlations in biological networks. This package was based on Fisher’s z-test and details were explained in (Fukushima, 2013; Zhou et al., 2021).

### Gene Enrichment Analysis

R package clusterProfiler was implemented to conduct enrichment analysis of clustered genes in blue and darkred modules. We used a hypergeometric distribution test for the classification of enrichment terms. And *p* values were adjusted by false discovery rate (FDR) method, the cutoff of which was set to be 0.05 (Yu et al., 2012).

### Gene Network Visualization

Cytoscape (3.9.0) was used to realize visualization of networks (Doncheva et al., 2019).

### Statistical Analysis

We applied Student’s *t*-test to identify genes differentially expressed between sepsis and healthy samples. *p* values were adjusted by the Benjamini–Hochberg method (Hochberg and Benjamini, 1990). Differentially expressed genes were defined as adjusted *p* value less than 0.05. We employed Fisher’s z-test to evaluate differential correlations of gene pairs between sepsis and healthy patients. And lfdr less than 0.05 was regarded as significant differential correlations.

## Results and Discussion

### Co-Expression Network Construction

Pearson’s correlation coefficient was applied to cluster samples from GSE134347. After removing outliers, a sample clustering tree was drawn (Figure 1B). Co-expression network was constructed from 25,245 coding and non-coding genes through WGCNA approach. We set soft-thresholding power five for satisfying scale-free topology of network, in which the corresponding R^2^ was 0.81 (Figure 1C). And we detected twenty-six modules in the network, as shown in a cluster dendrogram (Supplementary Material S1). The members in each module were listed in Supplementary Material S2. Apart from the grey module consisted of many un-classified members, orange module contained the minimum 33 genes, while the maximum 10,821 genes were included in turquoise module.

**FIGURE 2:**
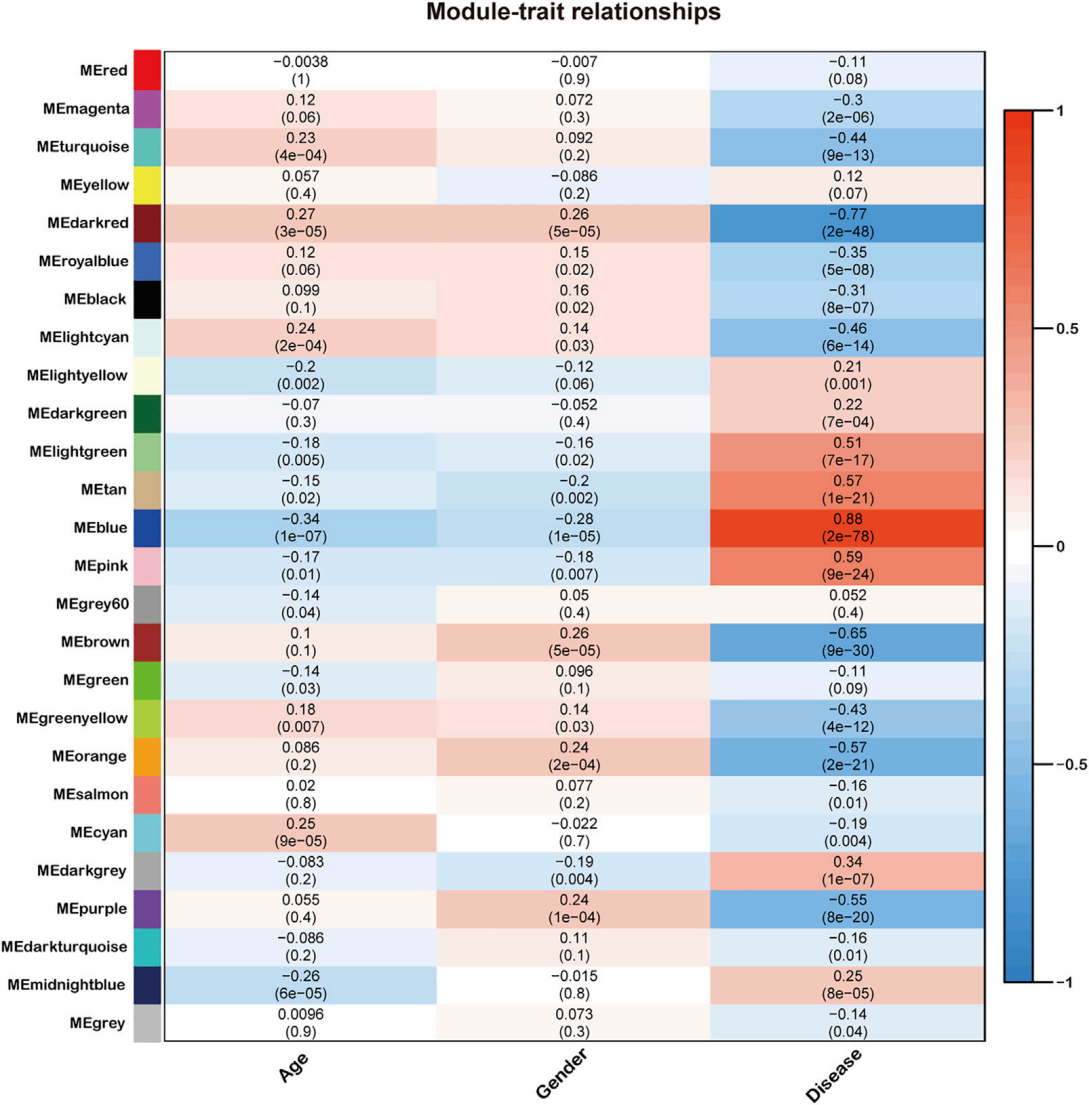
Identification of modules associated with the clinical traits of sepsis. Heatmap of the correlation between the module eigengenes and clinical traits of sepsis. All genes were clustered into twenty-six modules, of which each was labeled with one color.

Next, we quantified module-trait associations (Figure 2), in which the blue and darkred modules exhibited the most significant associations with sepsis. The corresponding correlation coefficients of blue and darkred modules were 0.88 (*p* = 2 × 10–78) and −0.77 (*p* = 2 × 10–48), respectively. In addition, GS and MM analysis demonstrated that genes highly significantly associated with sepsis were also the most crucial factors of modules associated with sepsis (Supplementary Material S3).

### Module Genes Enrichment

**FIGURE 3:**
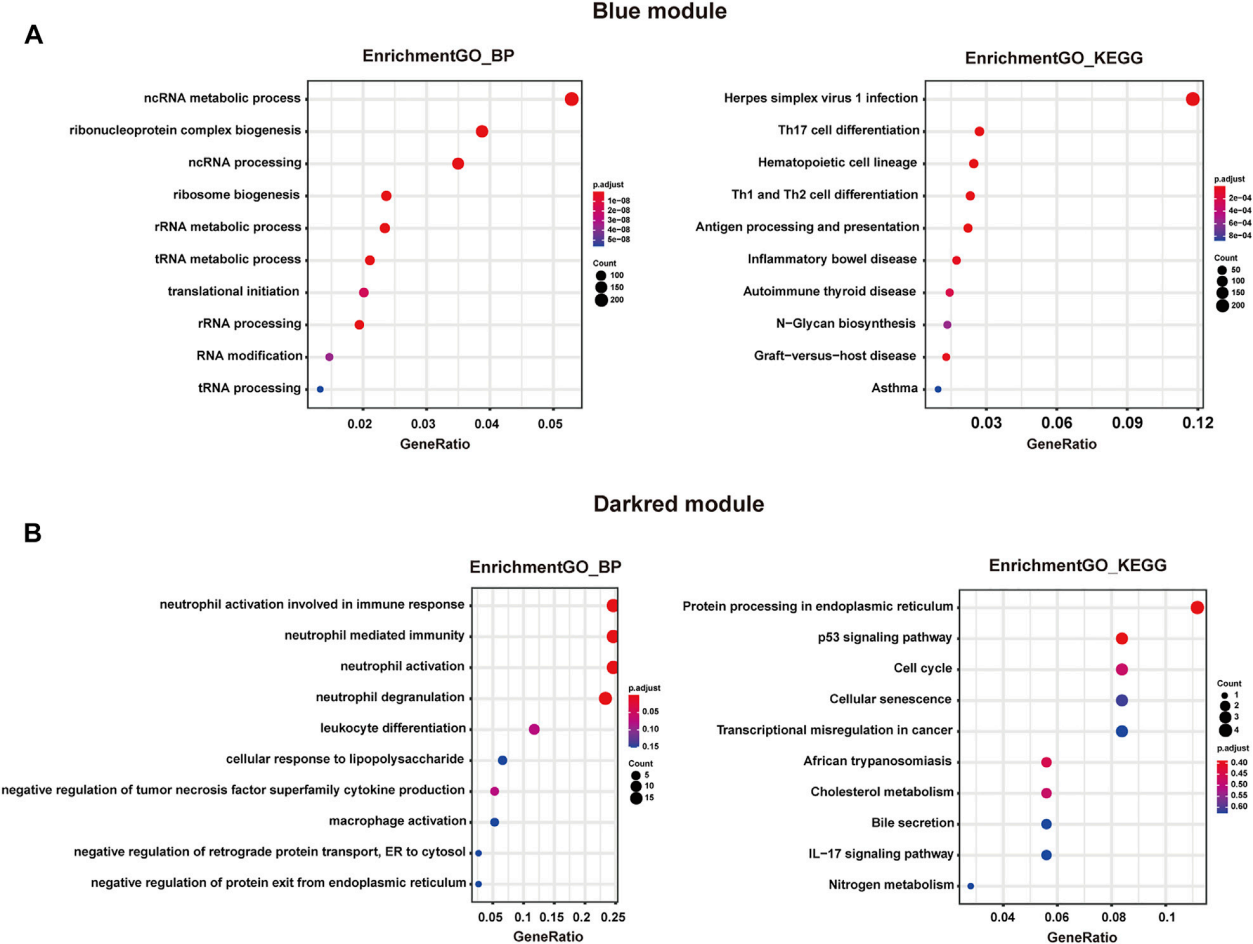
Functional enrichment analysis of genes in the blue and darkred modules. **(A)** Left panel: GO analysis showed top ten enriched biological processes in blue module. Right panel: KEGG analysis showed top ten enriched pathways in blue module. **(B)** Left panel: GO analysis showed top ten enriched biological processes in darkred module. Right panel: KEGG analysis showed top ten enriched pathways in darkred module.

Then, Gene Ontology (GO) and Kyoto Encyclopedia of Genes and Genomes (KEGG) enrichment analysis of these two modules were performed. As shown in Figure 3A, genes in the blue module were significantly enriched in ncRNA metabolic process, Herpes simplex virus 1 infection, and Th17 cell differentiation. Notably, as a less well studied subset of CD4^+^ Th cells, reduced Th17 cell responses have been observed in sepsis on account of low expression levels of involved transcription factors, bringing about increased susceptibility of patients to secondary fungal infections (Monneret et al., 2011; Hotchkiss et al., 2013). Our study provided additional evidence on the pivotal roles of Th17 cell function in sepsis. As shown in Figure 3B, genes in the darkred module were enriched in neutrophil activation involved in immune response and neutrophil degranulation. Consistent with previous studies, abnormal behaviors of neutrophil including delayed apoptosis have been witnessed during the early stage of sepsis (Drifte et al., 2013). Meanwhile, the most severely reduced neutrophil function has been investigated in patients with the highest risk of acquiring nosocomial infections (Nedeva, 2021), bestowing the importance of neutrophil responses on sepsis occurrence and progression.

### Differential Correlations Identification

**FIGURE 4:**
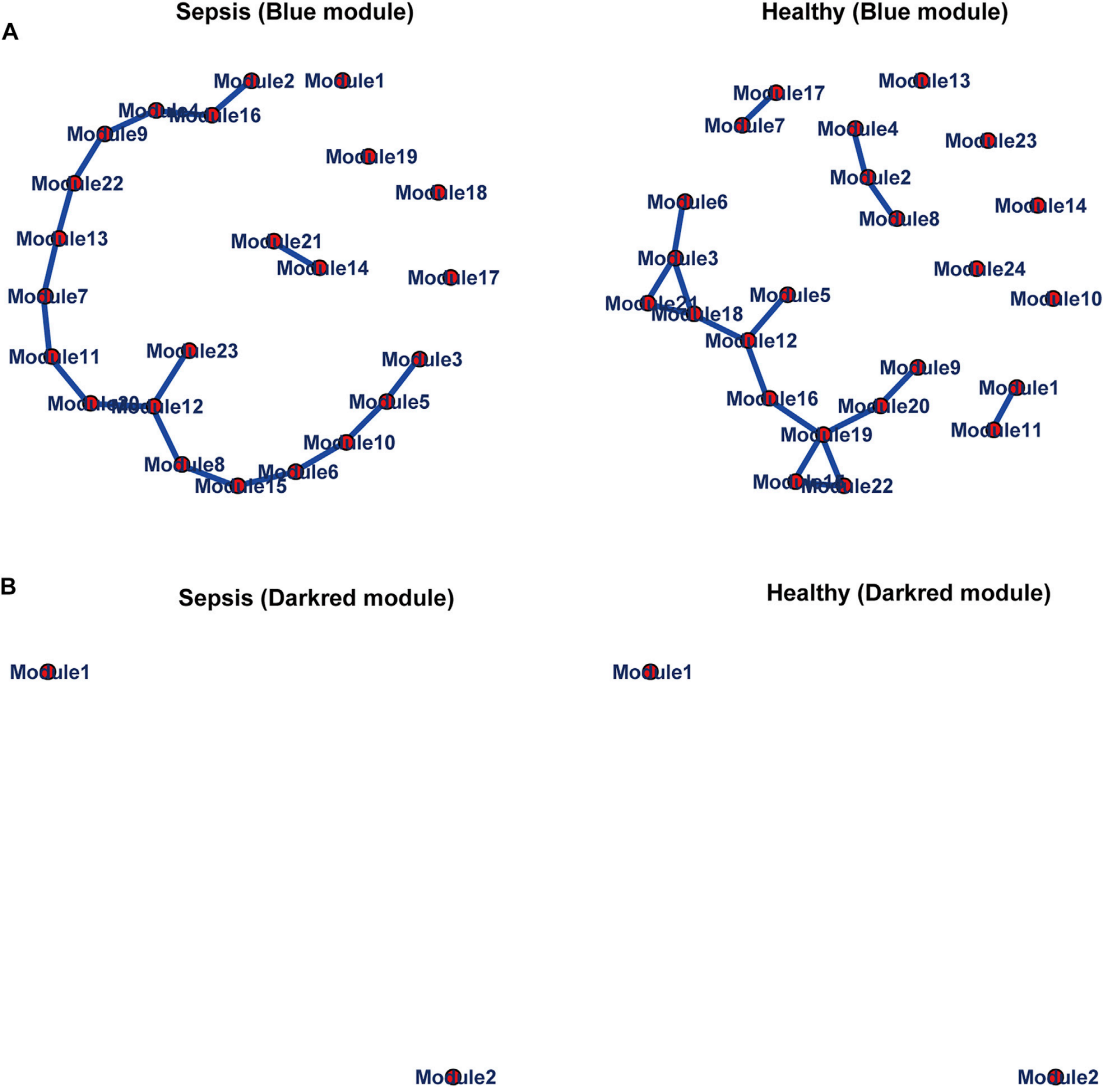
Module networks. The blue **(A)** and darkred **(B)** module networks from GSE134347 were shown. Each node represented one module. Each edge represented module correlation.

We further chose genes in the blue and darkred modules to estimate differential correlations, which were grouped based on expression patterns in each subtype (sepsis or healthy) under the *cluster. molecule* function of DiffCorr package. We utilized (1-correlation coefficient) as a distance measure based on *cutree* function. Two functions named *get. eigen.molecule* and *get. eigen.molecule.graph* were applied for module networks visualization (Figure 4). The *comp.2. cc.fdr* function offered the resultant pair-wise differential correlations among blue and darkred modules.

**FIGURE 5:**
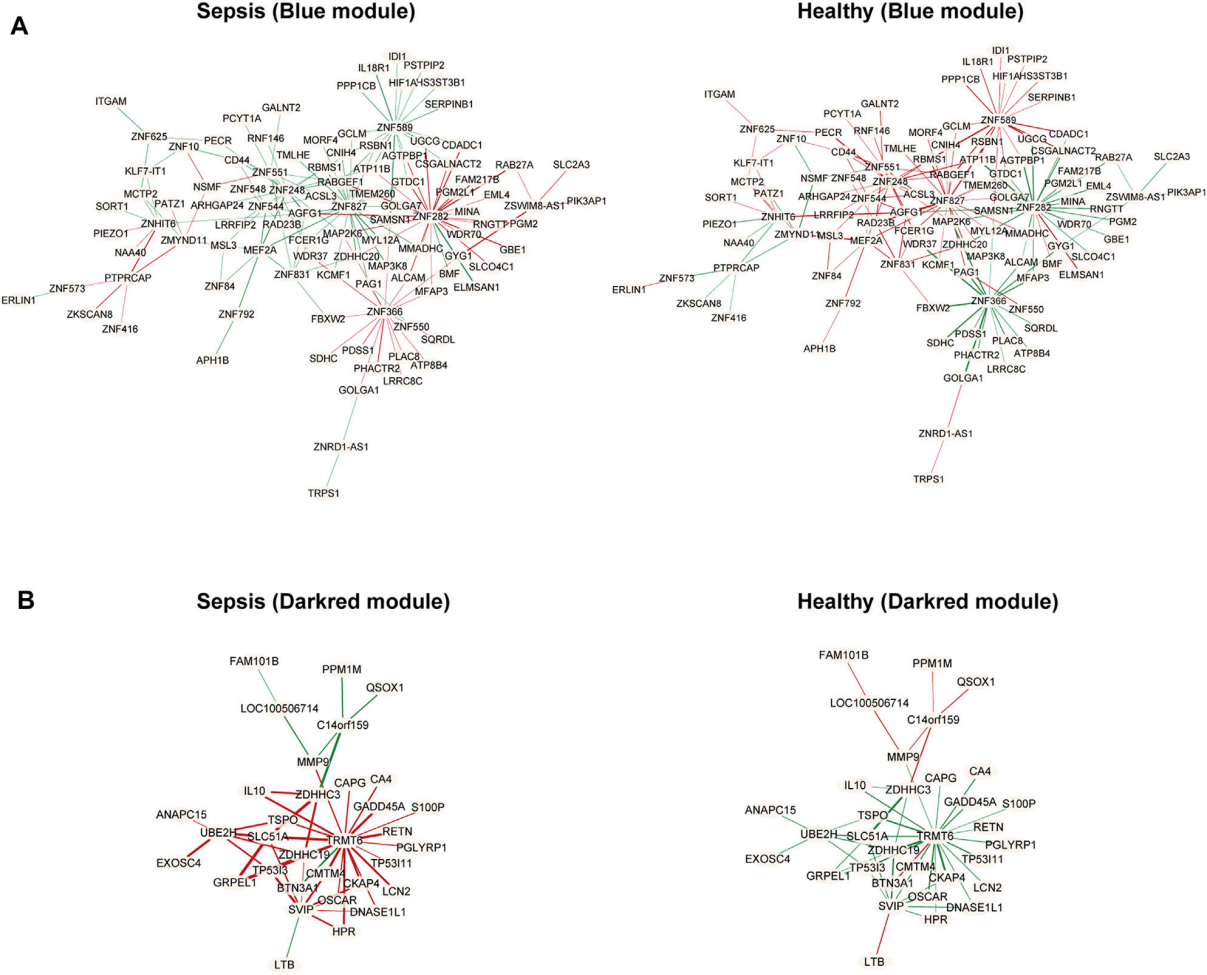
Differential co-expressed gene networks in the blue **(A)** and darkred **(B)** modules from GSE134347. Each node represented one gene. Each edge represented correlation between two genes, in which red meant positive correlation and green negative correlation. The thicker edge represented the stronger correlation coefficient.

R package DiffCorr also identified oppositely correlated pairs. For example, two genes positively correlated in sepsis tissues and negatively correlated in healthy tissues, or *vice versa*. These switched gene pairs were worthy noticed for their crucial roles in understanding molecular mechanisms in the progression of sepsis. Totally one hundred and seventy-four oppositely correlated gene pairs from the blue module and forty-nine from the darkred module were obtained (Supplementary Material S4), whose interaction networks were presented in Figure 5. The top ten significant differentially correlated gene pairs between sepsis and healthy tissues from blue and darkred modules were shown in Table 1.

**TABLE 1:** Top ten significant differentially correlated gene pairs from blue and darkred modules between healthy and sepsis tissues.

Molecule X	Molecule Y	r1 (Sepsis)	r2 (Healthy)	lfdr^a^ (Difference)	Module color
ZMYM6NB	FAM20A	0.406621495	−0.728163034	0	Blue
ZNF366	GOLGA1	0.410168886	−0.681188564	0	Blue
ZNF609	FAM20A	0.42304016	−0.680428116	0	Blue
ZNF366	WDR37	0.443006043	−0.647605336	0	Blue
ZNF366	FBXW2	0.401904401	−0.643995081	0	Blue
ZNF282	ATP11B	0.422093972	−0.63235805	0	Blue
ZNF366	SDHC	0.418454554	−0.626207078	0	Blue
ZNF366	PAG1	0.436699597	−0.610998009	0	Blue
ZNF366	MAP2K6	0.427097546	−0.609906763	0	Blue
ZNF282	AGTPBP1	0.512056621	−0.592121258	0	Blue
ZDHHC3	C14orf159	−0.709976288	0.51884098	0	Darkred
TRMT6	BTN3A1	−0.57700653	0.498814377	0	Darkred
TRMT6	OSCAR	0.519497637	−0.537521238	0	Darkred
TRMT6	CA4	0.564335391	−0.491940678	0	Darkred
TSPO	TRMT6	0.570633674	−0.551005684	0	Darkred
ZDHHC19	UBE2H	0.584905223	−.452219991	0	Darkred
ZDHHC19	TRMT6	0.586845831	−0.641186062	0	Darkred
SVIP	CKAP4	0.603948148	−0.440800517	0	Darkred
SVIP	CMTM4	0.632331442	−0.468788719	0	Darkred
ZDHHC3	IL10	0.635678135	−0.408650989	0	Darkred

a Local FDR.

### Biological Analysis of Hub Genes With Differential Correlations

**FIGURE 6:**
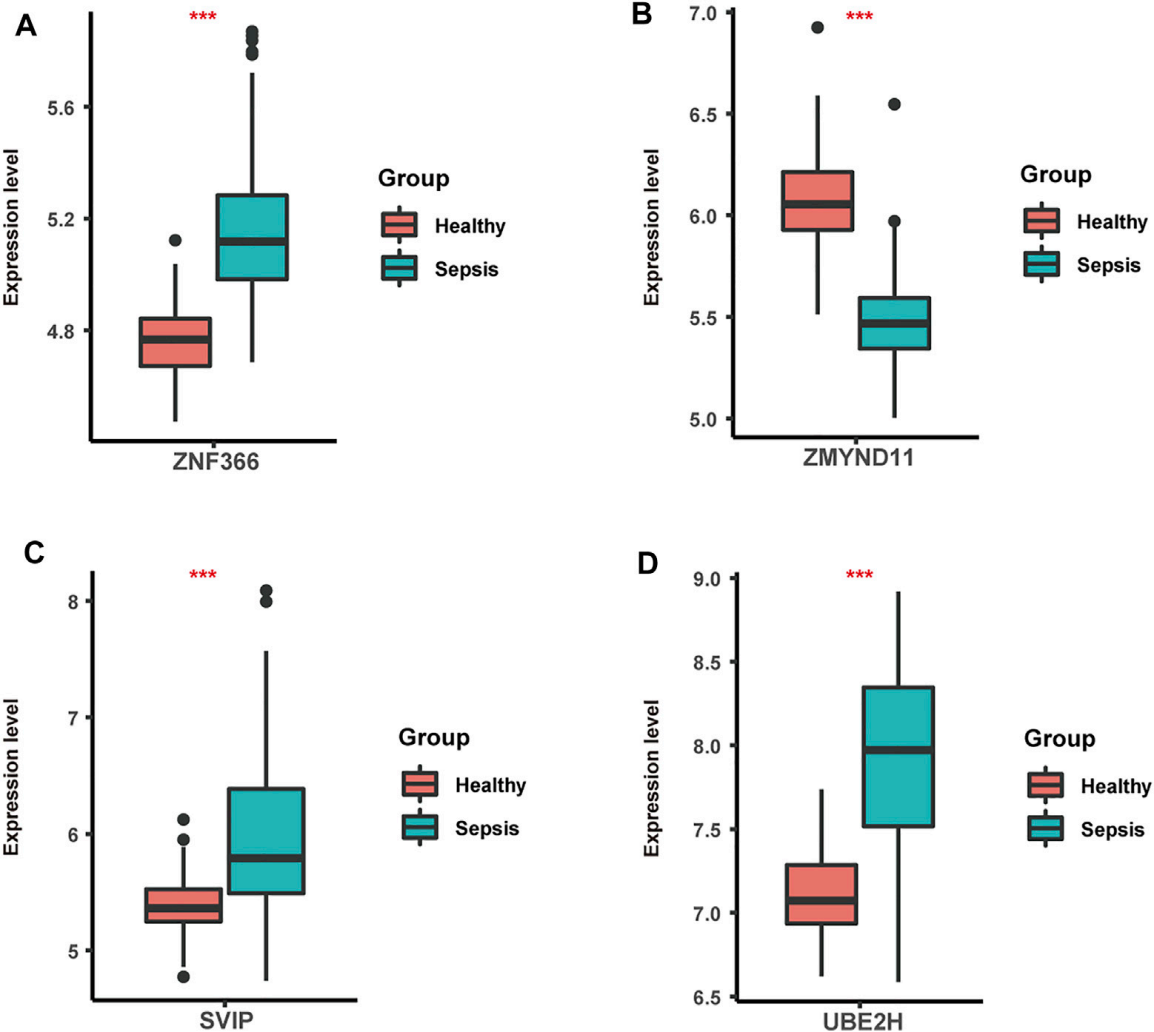
The expression levels of ZNF366 **(A)**, ZMYND11**(B)**, SVIP **(C)** and UBE2H **(D)** in sepsis and healthy samples from GSE134347. These four hub genes were all differentially expressed. ^***^ *p* < 0.001.

Lastly, we focused on four hub genes acting as master regulators due to possessing most links with other genes. Zinc finger protein 366 (ZNF366), also known as DC-SCRIPT, belongs to the zinc ring finger protein family and has recently been reported to regulate dendritic cell development (Søndergaard et al., 2015; Wang et al., 2018). ZNF366 has been shown to suppress toll like receptor-mediated expression of IL-10 through modulating NF-κBp65 activation (Søndergaard et al., 2015). Decreased numbers and disabilities of dendritic cells have been widely observed in sepsis followed by immune responses alterations (Roquilly and Villadangos, 2015; Venet and Monneret, 2017; Wang et al., 2020). Additionally, sepsis-induced dendritic cell blockade has been reported to prevent mice from sepsis-induced death (Meng et al., 2017). We also observed its elevated levels in sepsis compared to healthy samples (Figure 6A). Given the great importance of ZNF366 in coordinating dendritic cell function, our study expanded its roles in sepsis progression which needed further validations.

Another zinc finger protein that takes the central position in the differentially co-expressed gene network is MYND-type containing 11 (ZMYND11), which is the negative regulator of NF-κB signaling and vastly impacts the replication of various viruses including Hendra virus and Epstein-Barr virus (Ikeda et al., 2010; Stewart et al., 2013). As previously described, numerous patients with sepsis witnessed the activation of NF-κB pathway initiated by pathogen-associated molecular pattern or danger-associated molecular pattern, emphasizing its great contributions during sepsis (Hayden et al., 2006; Chen et al., 2019; Wang et al., 2019). In line with it, significant lower levels of ZMYND11were detected in sepsis than healthy samples (Figure 6B). Also, impaired NF-kB activation has been correlated with sepsis-induced acute lung injury (Chen et al., 2021). Thus, it could be inferred that ZMYND11 participated in sepsis progression through NF-κB signaling and further mechanisms needed to be explored.

Small p97/VCP-interacting protein (SVIP), localized to the ER membrane by myristoylation, is highly expressed in central nervous system and related to autophagy modulation (Wu et al., 2013; Jia et al., 2019). Further mechanisms have demonstrated that overexpression of SVIP protected hepatocytes from the toxicity of CCL_4_ through enhancing LC3 lipidation and activating autophagy (Jia et al., 2019). Prior studies have also found its essential roles in lysosomal dynamic stability and autophagosomal-lysosomal fusion (Johnson et al., 2021). Autophagy dysregulation has been observed in organ injury induced by sepsis, in which autophagy exerted vital effects on programmed cell death pathway and inflammation (Lo et al., 2013; Qiu et al., 2018; Zhao et al., 2019). Meanwhile, animal experiments have endowed autophagy with protective roles in septic brain injury (Su et al., 2015). Also, higher levels of SVIP were observed in sepsis compared to healthy samples (Figure 6C). Based on the above evidence, our study firstly illustrated the underlying roles of SVIP in sepsis through autophagy-related pathways.

Firstly identified in yeast and human placenta, ubiquitin conjugating enzyme E2 H (UBE2H) belongs to the structurally and functionally conserved family of E2s and is involved in ubiquitination and proteasome-mediated protein degradation and regulated by TNF-α signaling (Kaiser et al., 1994; Li et al., 2003; Clague et al., 2015). Increasing evidence has indicated the potential roles of UBE2H in human brain diseases such as amyotrophic lateral sclerosis, Alzheimer’s disease and autistic disorder (Martin et al., 2009; Sokolowski et al., 2018; Lim and Joo, 2020). Nevertheless, there is scant report on the links between UBE2H and sepsis. Previous data has shown that body protein loss during sepsis was caused by upregulation of ubiquitin genes and ubiquitin-proteasome pathway (Hasselgren and Fischer, 1997). Moreover, as a hypoxia-mediated gene, UBE2H may be speculated to participate in tissue hypoxia in septic shock (Rello et al., 2017). Here, we also found elevated levels of UBE2H in sepsis compared to healthy samples (Figure 6D). Therefore, our study firstly revealed the function of UBE2H in sepsis, offering experimental clues for further investigations.

## Conclusion

As the preliminary steps toward genetic regulatory networks, gene correlation approaches offered clues about uncovering function of mysterious genes. In this study, WGCNA and DiffCorr were employed to find out novel hub genes including ZNF366, ZMYND11, SVIP and UBE2H, and we proposed for the first time their causative factors during sepsis progression. Although biological analysis proved their vital roles in understanding pathogenesis of sepsis, these genes were not confirmed experimentally. Next we plan to integrate more datasets and conduct functional experiments including loss-of-function to underline mechanisms explaining their ability to trigger abnormal host response to infection.

## Data Availability

The datasets presented in this study can be found in online repositories. The names of the repository/repositories and accession number(s) can be found in the article/Supplementary Material.
