# Supplementary material for: Identification of Hub Genes With Differential Correlations in Sepsis
Source: Front Genet. 2022 Mar 24;13:876514. doi: 10.3389/fgene.2022.876514 (PMC8987114; doi:10.3389/fgene.2022.876514)
Supplement: Supplementary file 1 [file DataSheet2.PDF]

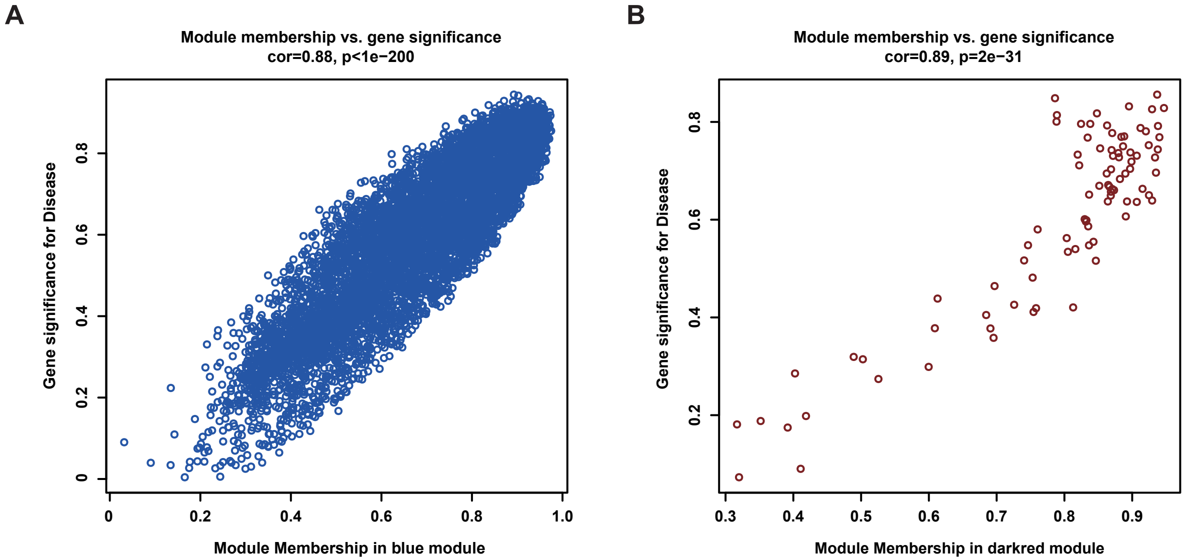
A
Module membership vs. gene significance
cor=0.88, p<1e−200
Gene significance for Disease
0
0.2
0.4
0.6
0.8
0
0.2
0.4
0.6
0.8
1.0
Module Membership in blue module
B
Module membership vs. gene significance
cor=0.89, p=2e−31
Gene significance for Disease
0.2
0.4
0.6
0.8
0.3
0.4
0.5
0.6
0.7
0.8
0.9
Module Membership in darkred module
