# Supplementary material for: Identification of Hub Genes With Differential Correlations in Sepsis
Source: Front Genet. 2022 Mar 24;13:876514. doi: 10.3389/fgene.2022.876514 (PMC8987114; doi:10.3389/fgene.2022.876514)
Supplement: Supplementary file 4 [file DataSheet1.PDF]

Gene dendrogram and module colors in block 1

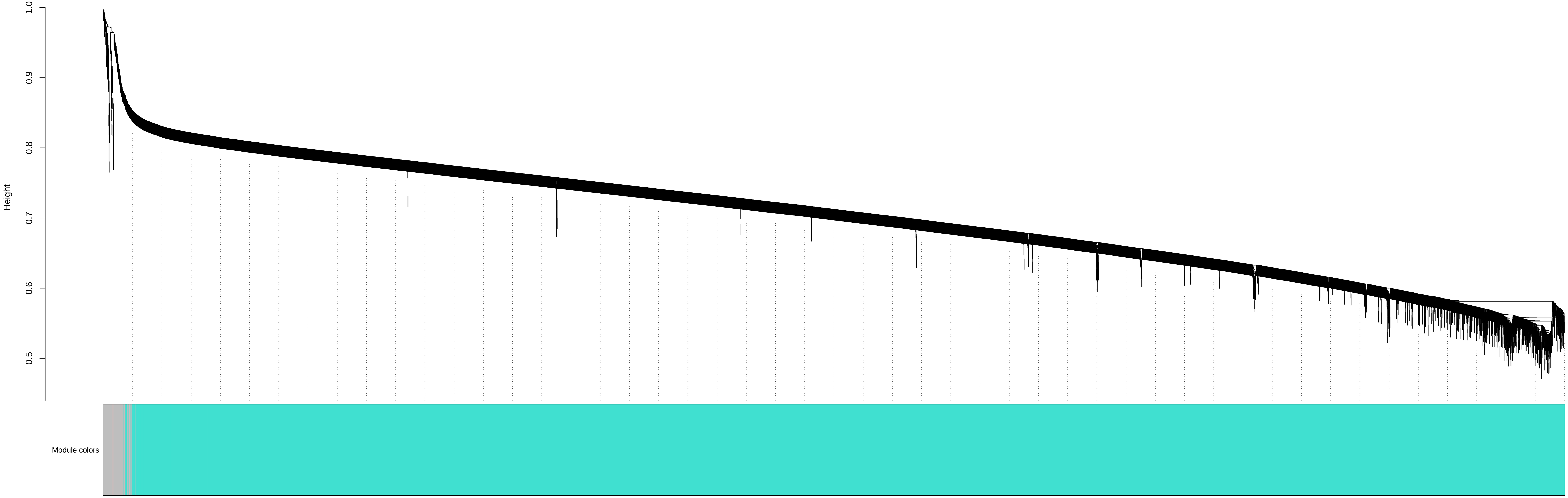

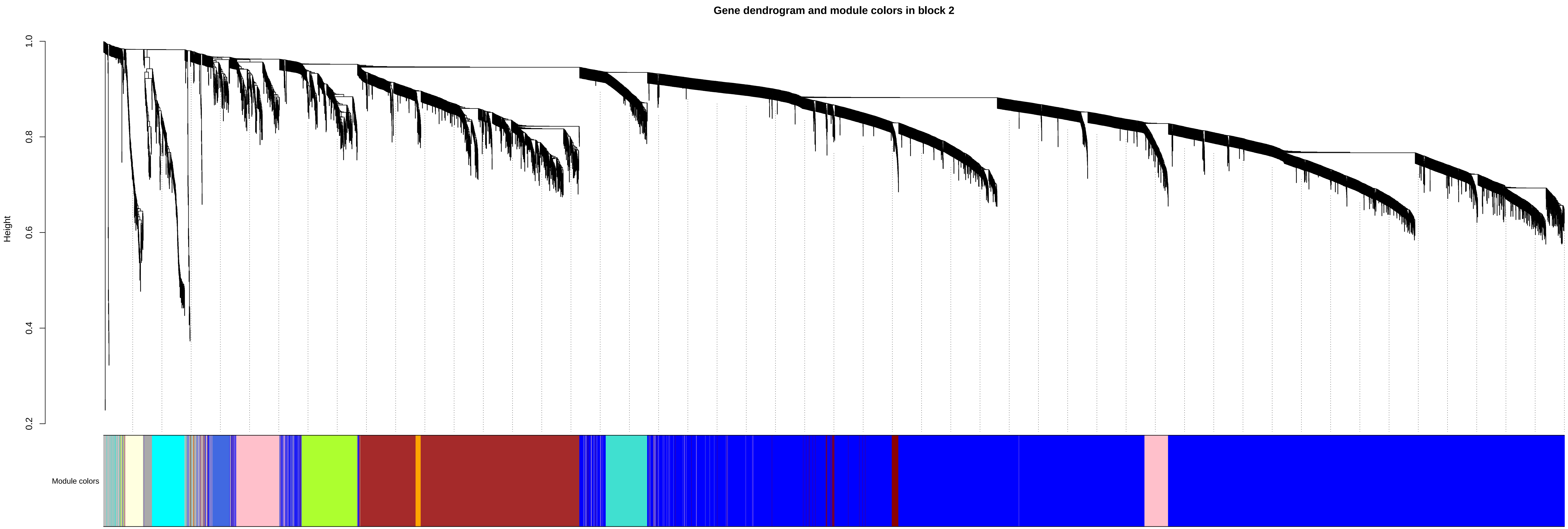
Gene dendrogram and module colors in block 2
Height
1.0
0.8
0.6
0.4
0.2
Module colors

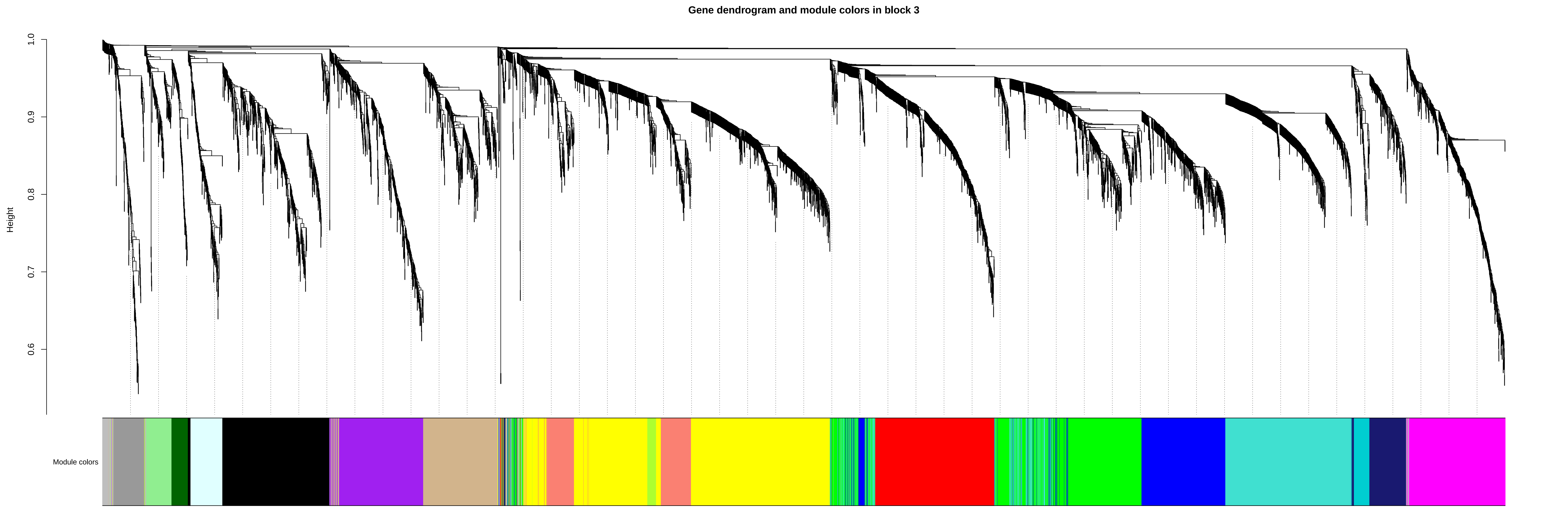
Gene dendrogram and module colors in block 3
Height
1.0
0.9
0.8
0.7
0.6
Module colors
